# Risk factors for adult interpersonal violence in suicide attempters

**DOI:** 10.1186/1471-244X-14-195

**Published:** 2014-07-07

**Authors:** Tomas Moberg, Marlene Stenbacka, Erik G Jönsson, Peter Nordström, Marie Åsberg, Jussi Jokinen

**Affiliations:** 1 The Department of Clinical Neuroscience/Psychiatry, Karolinska Institutet, Karolinska University Hospital, Solna, SE-171 76 Stockholm, Sweden; 2 Department of Public Health Sciences, Division of Social Medicine, Karolinska Institutet, SE-171 76 Stockholm, Sweden; 3 NORMENT, KG Jebsen Centre for Psychosis Research, Institute of Clinical Medicine, University of Oslo, Oslo, Norway; 4 Department of Clinical Sciences, Karolinska Institutet, Stockholm, Sweden

**Keywords:** Suicide, Interpersonal violence, Substance abuse, Aggression, Violent behaviour child

## Abstract

**Background:**

Suicidal and violent behaviours are interlinked and share common biological underpinnings. In the present study we analysed the association between violent behaviour as a child, childhood trauma, adult psychiatric illness, and substance abuse in relation to interpersonal violence as an adult in suicide attempters with mood disorders.

**Methods:**

A total of 161 suicide attempters were diagnosed with Structured Clinical Interviews and assessed with the Karolinska Interpersonal Violence Scale (KIVS) measuring exposure to violence and expressed violent behaviour in childhood (between 6-14 years of age) and during adult life (15 years or older). Ninety five healthy volunteers were used as a comparison group. A logistic regression analysis was conducted with the two KIVS subscales, expressed violent behaviour as a child and exposure to violence in childhood together with substance abuse, personality disorder diagnoses and age as possible predictors of adult interpersonal violence in suicide attempters.

**Results:**

Violent behaviour as a child, age and substance abuse were significant predictors of adult interpersonal violence. ROC analysis for the prediction model for adult violence with the KIVS subscale expressed violence as a child gave an AUC of 0.79. Using two predictors: violent behaviour as a child and substance abuse diagnosis gave an AUC of 0.84. The optimal cut-off for the KIVS subscale expressed violence as a child was higher for male suicide attempters.

**Conclusions:**

Violent behaviour in childhood and substance abuse are important risk factors for adult interpersonal violent behaviour in suicide attempters.

## Background

Suicidal and violent behaviors are interlinked through behavioral dysregulation of aggression reflecting reduced central serotonergic activity [1]. Repeaters of violent offences had a twofold higher suicide risk even after controlling for psychiatric inpatient care in a recent longitudinal population based study [2], whereas, in a cross-sectional study of suicidal inpatients, more than 90% reported that they had exposed their partner for violence in the past year [3]. Thus, not only suicide risk assessment but also violence risk assessment is important in the clinical management of suicide attempters.

Affective disorders are very common among suicide attempters, as well as a high degree of comorbidity with substance abuse and personality disorders. It has been argued that there is a significant, yet modest relationship, between severe mental illness such as affective disorders and violence [4]. Furthermore, there seems to be a significantly increased risk of violence when taking all types of personality disorders into account [5]. However, higher incidence of violence in patients with severe mental illness such as bipolar disorder and major depression can be largely explained by other factors associated with violence, such as previous violence and substance abuse [6].

Even though the psychiatric comorbidity is so prevalent, there is a paucity of studies on the impact of psychiatric comorbidity patterns on violence risk in suicide attempters with mood disorders.

While many studies have reported a positive association between maltreatment in childhood and criminality/violent behavior in adulthood, others have failed to detect such an association.

The cycle of violence - the exposure to physical abuse in childhood and violent behavior in adulthood - has been often reported [7]. A review of scientific longitudinal studies concluded that physical abuse in childhood predicted violent criminal offending later in life [8]. However, childhood maltreatment more often leads to different trajectories, with higher risk of psychiatric illness and suicidal behavior as an adult.

The association between childhood disruptive behaviors and adult violent behavior seems to be more robust. Frequent bullying among boys had an independent effect on violent criminality that went beyond childhood psychopathology in a Finnish longitudinal study [9]. To our knowledge, no earlier studies have assessed the impact of both childhood maltreatment and childhood violent behavior on used interpersonal violence as an adult in well characterized clinical populations of suicide attempters taking into account adult psychopathology and the comorbidity patterns.

The aim of the present study was to analyse the association between childhood trauma, violent behaviour as a child and Axis I and Axis II psychiatric diagnoses according to the Diagnostic and Statistical Manual of Mental disorders, third revised (DSM-III-R) and fourth editions (DSM-IV) in relation to self-reported interpersonal violence as an adult in suicide attempters. Since we used a clinical rating scale to assess both expressed violence and exposure to violence as a child, we also tested optimal thresholds of the scale expressed violence as a child to predict adult interpersonal violence. We hypothesized that high degree of psychiatric comorbidity would be associated with adult violent behaviour. We also hypothesized that both expressed violence as a child and exposure to violence in childhood would be independent risk factors for violent behaviour as an adult. Since many studies of suicidal and violent behaviour have shown gender differences [10,11], we assessed men and women separately.

## Methods

### Study setting

Patients having their clinical follow-up after a suicide attempt at the Suicide Prevention Clinic at the Karolinska University Hospital and who participated in two studies of biological and psychological risk factors for suicidal behaviour. The Regional Ethical Review Board in Stockholm approved the study protocols (Dnr 93-211 and Dnr 00-194) and the participants signed informed consent forms.

### Participants

A total of 161 suicide attempters (63 men and 98 women) for whom the Karolinska Interpersonal Violence Scale (KIVS) ratings had been performed were enrolled in the study. Inclusion criteria were a recent suicide attempt (within one month), fair capacity to communicate verbally and in writing in the Swedish language and an age of 18 years or older. Exclusion criteria were schizophrenia spectrum psychosis, intravenous drug abuse, dementia and mental retardation. The cohort and the Karolinska Interpersonal Violence Scale have been described in details in another study [12]. Patients were recruited between years 1993 to 2005 from the catchment area of the psychiatric clinic at the Karolinska University Hospital. During the first study period (1993-1998) the exact information of the sampling procedure with participation rate was not registered. During the second study period (2000-2005), 258 patients (169 women and 89 men) from the catchment area made a suicide attempt and came into contact with the Suicide Prevention Clinic. Sixty-one patients were excluded due to exclusion criteria above, 50 patients did not want to participate in the study and 47 patients were not proposed to participate due to reasons like early refusal to have a clinical follow-up, holiday period, or moving to another part of the country. A total of 100 suicide attempters (67 women and 33 men) were enrolled during the second study period.

The mean age of patients was 35 years (SD = 12.1; range 18-69). The mean age did not differ between men and women. Thirty-two patients (20%) had used a violent suicide attempt method (14 women, 14.3% and 18 men, 28.6%). A suicide attempt was defined as a self-destructive act with some degree of intent to die and the suicide attempt method was defined as violent or not according to the criteria of Träskman et al [13].

### Healthy volunteers

**Figure 1:**
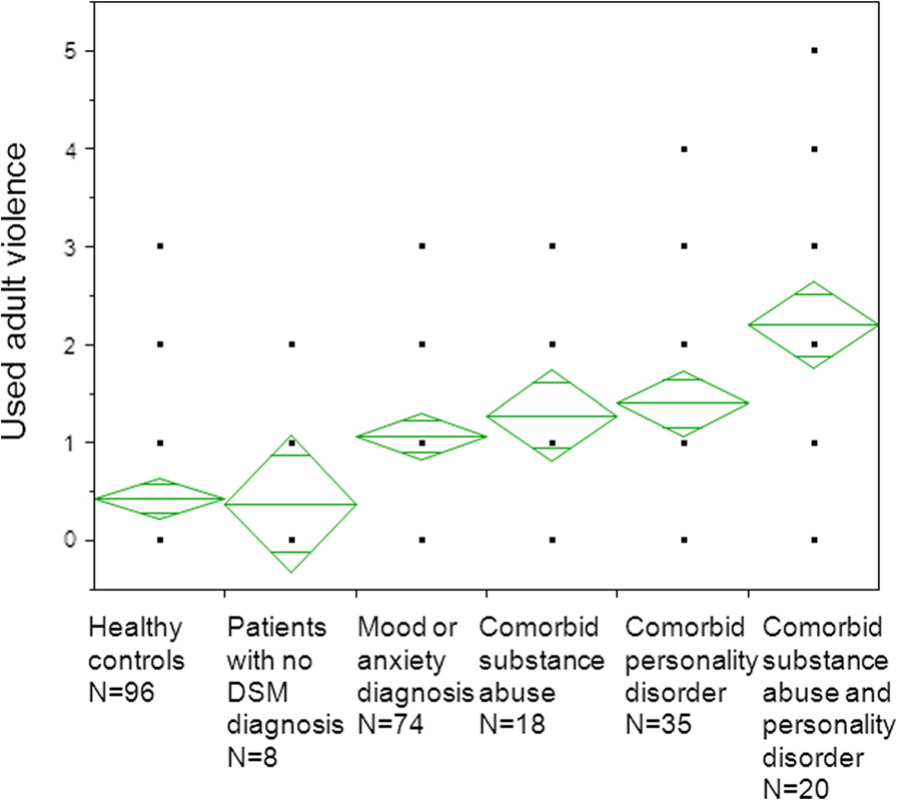
**Levels of expressed interpersonal violence as an adult in healthy controls (n = 95) and in suicide attempters (n = 161) divided in groups with regard to DSM diagnoses.** Six patients were not diagnosed for Axis II and were not included in the analysis. Diagnostic groups were not overlapping. The upper, middle and lower vertical lines of the rhomboids show the mean and the standard error. The black squares show KIVS ratings represented in each sub-sample. Healthy controls differed from suicide attempters with Axis I or II diagnoses. Suicide attempters with mood or anxiety diagnosis as well as both comorbid personality disorder and substance abuse reported the highest levels of adult interpersonal violence.

Ninety-five healthy volunteers (38 men and 57 women) were recruited in Stockholm 2003-2004. They were screened by a psychiatrist to verify the absence of current mental disorder. The mean age for healthy volunteers was 40 years (SD = 11, range 18-63). This comparison group was only included in Figure 1.

### Instruments

To establish diagnoses according to (DSM-III-R and DSM-IV), the participants were interviewed by a trained psychiatrist using the research version of the *Structured Clinical Interview for DSM-III-R or DSM-IV, Axis I (SCID I)*[14,15]. Trained clinical psychologists established Axis II, i.e. personality disorder diagnoses with a *SCID II* interview [16,17].

Ninety-five percent of the suicide attempters had at least one current Axis I or II psychiatric diagnosis.

Of the patients 78% fulfilled criteria for mood disorders (unipolar, major depressive disorder, single episode or recurrent, bipolar disorder, depressed or dysthymic disorder), five percent for adjustment disorder and five percent for anxiety disorders (half of them with post-traumatic stress disorder). Three percent of the patients had a substance related disorder as their major diagnosis, but 25% of the patients had a comorbid lifetime diagnosis of substance related disorder (83% alcohol dependence). *SCID II interviews* were not performed in six patients. Among Axis II diagnoses, 55 patients (36%) fulfilled criteria for a personality disorder, 42% of them in Cluster B. Most patients with personality disorder had a diagnosis of borderline personality disorder (n = 19) or personality disorder not otherwise specified (n = 20). Eleven patients fulfilled criteria for a conduct disorder during childhood and seven patients for antisocial personality disorder. Table 1 shows diagnostic grouping of suicide attempters with regard to the degree of comorbidity.

**Table 1:** Diagnostic grouping of suicide attempters

**DSM diagnostic groups**	**Number of suicide attempters**
No DSM diagnosis	8
DSM mood or anxiety Axis I diagnosis without comorbid substance abuse or personality disorder	74
DSM mood or anxiety Axis I diagnosis with comorbid substance abuse only	18*
DSM mood or anxiety Axis I diagnosis with comorbid personality disorder only	35*
DSM mood or anxiety Axis I diagnosis with both comorbid substance abuse and personality disorder	20*

*Four patients not fulfilling criteria for mood or anxiety diagnosis but only criteria for substance abuse and/or personality disorder were included in the groups 3, 4 and 5 respectively.

Six patients were not diagnosed for Axis II and were not included in the analysis. Diagnostic groups were not overlapping.

*The Karolinska Interpersonal Violence Scale (KIVS)* contains four subscales assessing both exposure to violence and expressed violent behaviour in childhood (between 6-14 years of age) and during adult life (15 years or older) [12]. The ratings are based on a semi structured interview. Interviews and ratings (0-5 for each subscale, total 20) were performed and assessed by trained clinicians. The KIVS scale and the dichotomized violence levels used in the statistical analyses are presented in Additional file 1. Ratings in the subscale Used interpersonal violence as an adult were dichotomized: non-violent patients (0, 1, 2) and violent patients (3, 4, 5).

### Data analysis

Initial analyses were carried out to evaluate skewness and kurtosis of the distributions with Shapiro-Wilk test. Tests of non-parametric correlations were performed using Spearman rho. Nonparametric statistics, including Kruskal-Wallis or Wilcoxon test were applied for between group comparisons.

Based on the results of univariate analysis, standard multivariate logistic regression analyses were conducted with the two KIVS ratings, exposure to violence as a child and expressed violence as a child, together with substance abuse diagnosis, personality disorder diagnosis and age as possible predictors of adult interpersonal violence. To be defined as violent as an adult, violence score 3 or above in the KIVS subscale expressed violence as an adult was applied. Since many studies of violent and suicidal behaviour have shown gender differences and there were gender differences in KIVS subscale expressed violence as a child, we stratified for men and women separately. An ad hoc receiver operating characteristics (ROC) analysis was used to find optimal thresholds for significant clinical predictors for adult violence. ROC curves and tables were created to establish the optimal cut-off values. ROC areas under the curves (AUCs) were calculated as a measure of the diagnostic performance. The cut-off point that optimized sensitivity (proportion of violent patients correctly identified) and specificity (proportion of non-violent patients correctly identified) was applied. Pearson Chi-square and Fisher’s exact test were used for cross tabulations of categorical variables.

The *P* value was set at <0.05. The Statistical Package JMP 9 software, SAS Institute inc., Cary, NC, USA was used for all statistical analyses.

## Results

### Psychiatric diagnosis, comorbidity, and expressed interpersonal violence as an adult

Patients reported significantly more used adult violence compared to healthy controls (p < 0.0001, Kruskal-Wallis). Ninety-five percent of suicide attempters fulfilled criteria for at least one DSM diagnosis (Axis I or II). These patients reported significantly more adult interpersonal violence compared to suicide attempters without any DSM diagnosis (n = 8; p = 0.03, Wilcoxon test). Figure 1 shows group comparisons between healthy controls and patients divided in diagnostic categories concerning degree of comorbidity and used interpersonal violence as an adult (p < 0.0001, Kruskal-Wallis). Suicide attempters with both comorbid substance abuse and personality disorder had significantly higher scores of used adult violence compared to suicide attempters with only mood or anxiety disorder (p = 0.0025 Wilcoxon test) and suicide attempters with comorbid personality disorder (p = 0.047, Wilcoxon test).

### Childhood violence as a risk factor for adult violence in suicide attempters

Used interpersonal violence as a child was significantly correlated with adult interpersonal violence in suicide attempters (rho = 0.36, p < 0.0001). Exposure to violence as a child was also significantly correlated with used adult violence in suicide attempters (rho = 0.28, p = 0.0003). Male patients rated higher compared to women in used violence during childhood (p < 0.0001, Kruskal-Wallis). There were no gender differences in ratings of exposure to violence as a child or in used violence as an adult among patients. Used interpersonal violence as a child was significantly correlated with adult interpersonal violence in both male and female suicide attempters (rho = 0.47, p < 0.0001 and rho = 0.38, p < 0.0001, respectively). Exposure to violence as a child was also significantly correlated with adult interpersonal violence in both male and female suicide attempters (rho = 0.32, p = 0.01 and rho = 0.24, p = 0.02, respectively).

### Regression analysis

A multivariate logistic regression analysis was conducted with the two KIVS ratings, i.e. used violence and exposure to violence as a child together with substance abuse diagnosis, personality disorder diagnosis and age as predictors of adult interpersonal violence in suicide attempters. The regression model was significant (Chi square = 45.6, DF = 5, p < 0.0001). Expressed violence as a child, substance abuse and age were significant predictors of adult interpersonal violence. Table 2 show odds ratios of clinical predictors of adult interpersonal violence. Broken down by gender, personality disorder predicted violence as an adult in male suicide attempters in Table 3.

**Table 2:** Predictors for violence as an adult in a sample 161 suicide attempters

	**Adult violence**	
	**Odds ratio (95% CI)**	**p value**
Expressed violence as a child	2.88 (1.54-6.03)	0.0009
Exposure to violence as a child	1.27 (0.82-1.99)	0.29
Substance abuse diagnosis	10.93 (3.36-40.74)	<0.0001
Personality disorder diagnosis	2.47 (0.74-8.70)	0.14
Age	0.91 (0.84-0.97)	0.0016

Multivariate logistic regression.

(Model fit: Chi-square = 45.6, DF = 5, p < 0.0001).

**Table 3:** Predictors for violence as an adult in female and male suicide attempters

	**Adult violence**			
	**Women (n = 98)**		**Men (n = 63)**	
	**Odds ratio (95% CI)**	***p *** **value**	**Odds ratio (95% CI)**	***p *** **value**
Expressed violence as a child	2.53 (0.84-8.27)	0.095	3.30 (1.16-13.10)	0.025
Exposure to violence as a child	1.26 (0.71-2.27)	0.42	1.60 (0.68-4.32)	0.28
Substance abuse diagnosis (yes vs no)	8.21 (1.66-49.85)	0.0096	22.05 (2.97-351.53)	0.0016
Personality disorder diagnosis (yes vs no)	1.02 (0.17-5.45)	0.98	13.94 (1.50-337.44)	0.018
Age	0.91 (0.82-0.98)	0.016	0.85 (0.70-0.97)	0.013

Women Model fit: Chi-square = 17.8, DF = 5, p = 0.0031. Men Model fit: Chi-square = 30.5, DF = 5, p < 0.0001.

### Receiver operating characteristic analysis

When the KIVS subscale expressed violence as a child was entered as a predictor for adult interpersonal violence among all the suicide attempters, a ROC analysis revealed AUC 0.79, sensitivity 100%, specificity 49% and optimal cut-off 1. Entering two predictors, i.e. expressed violence as a child and substance abuse diagnosis on the same sample, the ROC analysis showed an AUC of 0.84, a sensitivity of 71% and a specificity of 89%.

When the KIVS subscale expressed violence as a child was entered as a predictor for adult interpersonal violence among male suicide attempters, a ROC analysis revealed AUC 0.81, sensitivity 64%, specificity 88% and optimal cut-off 2. Entering two predictors, i.e. violent behaviour as a child and substance abuse diagnosis among male suicide attempters, the ROC analysis gave an AUC of 0.86, a sensitivity of 81% and a specificity of 84%.

When the KIVS subscale expressed violence as a child was entered as a predictor for adult interpersonal violence among female suicide attempters, a ROC analysis revealed AUC 0.78, sensitivity 100%, specificity 63% and optimal cut-off 1. Entering two predictors, i.e. violent behaviour as a child and substance abuse diagnosis among the female suicide attempters, revealed an AUC of 0.83, a sensitivity of 100% and a specificity of 52%.

## Discussion

In the present study, we investigated how early risk factors, i.e. violent behaviour as a child and exposure to interpersonal violence in childhood and current risk factors e.g. adult psychiatric comorbidity patterns were associated with adult interpersonal violence in a well characterized group of suicide attempters. The main finding was that violent behaviour as a child, age and substance abuse were significant independent predictors of adult interpersonal violence in suicide attempters.

Our finding that violent behaviour as a child predicted violence in adulthood is in line with other studies; prior studies have found that a history of any violent act and juvenile detention or a diagnosis of conduct disorder before age 15 predicted violent behaviour, even in relation to co-occurring severe mental illness and substance use [6,18]. When broken down by gender, violent behaviour as a child correlated significantly with violence as an adult in both men and women.

Not surprisingly, substance abuse was significantly associated with increased interpersonal violence as an adult. Several studies have shown that alcohol and drug abuse increases the risk of violence among patients with affective disorders [19-22]. In a population based study of the entire Swedish adult population during 12 years, 16% of all those who had been convicted for aggressive crimes had a history of contact with the health care system due to alcohol misuse. The corresponding figure for substance misuse was almost 12% [23]. In a longitudinal study over 30 years in New Zealand, those with many symptoms of alcohol abuse/dependence had a higher risk of becoming violent compared to those with few such symptoms [24].

When substance abuse was present together with a mood or anxiety disorder and a comorbid personality disorder, it was associated with a significant increased risk of violence as an adult. This increased risk appeared to be mediated by a personality disorder. This finding is supported by previous reports that borderline personality disorder with comorbid substance abuse elevates the risk for violence [25,26]. Some authors have suggested that alcohol is a trigger for aggression only for those who already have a tendency to react with aggression in conflicts [27]. This view is supported by a large study of nearly 8,000 college students who were enrolled at 38 sites around the world, where there was an association between binge drinking and partner violence, which, however, was wholly explained by antisocial traits or behaviors [28].

Comorbidity with personality disorder was associated with more interpersonal violence as an adult in male suicide attempters with mood or anxiety disorders and was an independent predictor for adult interpersonal violence. The most common personality disorder diagnoses were borderline personality disorder or personality disorder not otherwise specified. The present results are generally in line with the results of Yu and coauthors reporting a significantly increased risk of violence when taking all types of personality disorders into account [5]. The presence of confounding factors related to borderline personality disorder suggests caution in the interpretation of the literature concerning the connection between this condition and violence. A history of maltreatment during childhood, previous violence or criminality, comorbid psychopathy or antisocial personality seemed to be predictors of violence in patients with borderline personality disorder [29]. In our cohort six male patients had a comorbid antisocial personality disorder which may explain the association between personality disorders and used adult violence in men.

In our study, contrary to our hypothesis, exposure to violence as a child did not predict violence as an adult in multivariate logistic regression analyses. The literature contains some contradictory results. Findings from a cohort study indicated that those who had been abused or neglected as a child had an increased risk for delinquency, adult criminal behavior, and violent criminal behavior [7]. In another study there was a statistically significant correlation between the duration of being bullied and a greater number of reported aggressive behaviours, which allowed the researchers to conclude that there was an association between having been bullied in childhood and aggressive behavior in adulthood [30]. However, a recent prospective longitudinal study showed no association between poor treatment from ten to twelve years of age and non-violent or violent crimes between twelve to 24 years of age [31]. Furthermore, a previous study could not find any connection between the rougher forms of maltreatment during childhood and arrests in early adulthood [32].

Using ROC analysis for the prediction model for adult violence with expressed violence as a child we got an AUC of 0.79 for all suicide attempters. We correctly identified all 21 suicide attempters with proneness for adult violence; however the number of false positives was 71. The prediction model performed better in male suicide attempters, when including two significant predictors. Expressed interpersonal violence as a child and substance abuse gave an AUC of 0.86 and we identified correctly nine of eleven violent male patients while the number of false positives was eight. The optimal cut-off for used violence as a child was higher in males compared to female suicide attempters. Especially, the specificity was lower in the adult violence prediction models in female suicide attempters when including two significant predictors.

Following clinical implications are supported by the results of the study. Violent behavior in childhood should be measured in a structured way in violence risk assessments of suicide attempters. The subscale expressed interpersonal violence as a child of the Karolinska Interpersonal Violence Scale can be used to detect suicide attempters at risk for adult interpersonal violence. Subscale contains distinct levels of different type of violent behavior based on the severity of used interpersonal violence as a child. However, it is also important to assess substance abuse since the comorbidity with substance abuse clearly heightened the violence risk as an adult in both male and female suicide attempters. In men comorbid personality disorder was also associated with higher risk for adult interpersonal violence and especially male patients with both comorbid substance abuse and personality disorder evinced a high risk for adult violent behavior.

Strength of our study included diagnoses based on semi-structured SCID interviews and expressed violence measures obtained from semi-structured interviews. Furthermore, we had violence ratings from a healthy control group. Limitation of the study is its cross-sectional design, which prevents causal interpretations of the relationships. Furthermore, the sample size was rather small especially concerning some of the diagnostic subgroups. Another limitation was the absence of patients with intravenous drug abuse and schizophrenia, which were exclusion criteria, which means that the results are not generalizable to these groups.

## Conclusions

Since suicidal and violent behaviours are partly interlinked, it is important to do violence risk assessments in this patient group. Violent behaviour in childhood and substance abuse are important risk factors for interpersonal violent behaviour as an adult in suicide attempters. Violent behaviour in childhood, as well as substance abuse should be measured in a structured way as part of the clinical violence risk assessment in suicide attempters.

## Abbreviations

AUC: Area under the curve; DSM: The diagnostic and statistical manual of mental disorders, third revised (DSM-III-R) and fourth editions (DSM-IV); KIVS: the Karolinska Interpersonal Violence Scale; ROC: Receiver operating characteristics; SCID: Structured Clinical Interview for DSM-III-R or DSM-IV, Axis I (SCID I) and Axis II (SCID II).

## Competing interests

The authors declare that they have no competing interests.

## Authors’ contributions

MÅ created the KIVS, organized the original investigation and coordinated the collection of data. TM and JJ participated in the design of the study, performed the statistical analyses and drafted the manuscript. MS and EGJ contributed to the statistical analyses and revision of the manuscript. PN contributed to the revision of the manuscript. All authors read and approved the final manuscript.

## Pre-publication history

The pre-publication history for this paper can be accessed here:

http://www.biomedcentral.com/1471-244X/14/195/prepub

## Supplementary Material

Additional file 1 The Karolinska Interpersonal Violence Scale. Click here for file

## References

[B1] Moberg T Nordstrom P Forslund K Kristiansson M Asberg M Jokinen J CSF 5-HIAA and exposure to and expression of interpersonal violence in suicide attempters J Affect Disord 2011 132 1–2 173 178 21356560 10.1016/j.jad.2011.01.018

[B2] Stenbacka M Moberg T Romelsjo A Jokinen J Mortality and causes of death among violent offenders and victims–a Swedish population based longitudinal study BMC Public Health 2012 12 38 22251445 10.1186/1471-2458-12-38 PMC3329420

[B3] Heru AM Stuart GL Rainey S Eyre J Recupero PR Prevalence and severity of intimate partner violence and associations with family functioning and alcohol abuse in psychiatric inpatients with suicidal intent J Clin Psychiatry 2006 67 1 23 29 16426084 10.4088/jcp.v67n0104

[B4] Van Dorn R Volavka J Johnson N Mental disorder and violence: is there a relationship beyond substance use? Soc Psychiatry Psychiatr Epidemiol 2012 47 3 487 503 21359532 10.1007/s00127-011-0356-x

[B5] Yu R Geddes JR Fazel S Personality disorders, violence, and antisocial behavior: a systematic review and meta-regression analysis J Pers Disord 2012 26 5 775 792 23013345 10.1521/pedi.2012.26.5.775

[B6] Elbogen EB Johnson SC The intricate link between violence and mental disorder: results from the National Epidemiologic Survey on Alcohol and Related Conditions Arch Gen Psychiatry 2009 66 2 152 161 19188537 10.1001/archgenpsychiatry.2008.537

[B7] Widom CS The cycle of violence Sci (New York, NY) 1989 244 4901 160 166 10.1126/science.2704995 2704995

[B8] Murray J Farrington DP Risk factors for conduct disorder and delinquency: key findings from longitudinal studies Can J Psychiatry 2010 55 10 633 642 20964942 10.1177/070674371005501003

[B9] Sourander A Brunstein Klomek A Kumpulainen K Puustjarvi A Elonheimo H Ristkari T Tamminen T Moilanen I Piha J Ronning JA Bullying at age eight and criminality in adulthood: findings from the Finnish Nationwide 1981 Birth Cohort Study Soc Psychiatry Psychiatr Epidemiol 2011 46 12 1211 1219 21120451 10.1007/s00127-010-0292-1

[B10] Hawton K Sex and suicide. Gender differences in suicidal behaviour Br J Psychiatry 2000 177 484 485 11102320 10.1192/bjp.177.6.484

[B11] Swan SC Snow DL The development of a theory of women’s use of violence in intimate relationships Violence Against Women 2006 12 11 1026 1045 17043365 10.1177/1077801206293330

[B12] Jokinen J Forslund K Ahnemark E Gustavsson JP Nordstrom P Asberg M Karolinska Interpersonal Violence Scale predicts suicide in suicide attempters J Clin Psychiatry 2010 71 8 1025 1032 20797380 10.4088/JCP.09m05944blu

[B13] Traskman L Asberg M Bertilsson L Sjostrand L Monoamine metabolites in CSF and suicidal behavior Arch Gen Psychiatry 1981 38 6 631 636 6166274 10.1001/archpsyc.1981.01780310031002

[B14] First MB SR Gibbon M Williams JB Structured Clinical Interview for DSM-IV-TR Axis I Disorders, Research Version, Patient Edition (SCID-I/P) 2002 New York, NY: Biometrics Research, New York State Psychiatric Institute

[B15] Spitzer RL Williams JB Gibbon M First MB The Structured Clinical Interview for DSM-III-R (SCID). I: History, rationale, and description Arch Gen Psychiatry 1992 49 8 624 629 1637252 10.1001/archpsyc.1992.01820080032005

[B16] Farmer RF Chapman AL Evaluation of DSM-IV personality disorder criteria as assessed by the structured clinical interview for DSM-IV personality disorders Compr Psychiatry 2002 43 4 285 300 12107866 10.1053/comp.2002.33494

[B17] First MB SR Gibbon M Williams JB The Structured Clinical Interview for DSM-III-R Personality Disorders (SCID-II), part I: description J Pers Disord 1995 9 83 91

[B18] Fang X Massetti GM Ouyang L Grosse SD Mercy JA Attention-deficit/hyperactivity disorder, conduct disorder, and young adult intimate partner violence Arch Gen Psychiatry 2010 67 11 1179 1186 21041619 10.1001/archgenpsychiatry.2010.137

[B19] Fazel S Lichtenstein P Grann M Goodwin GM Langstrom N Bipolar disorder and violent crime: new evidence from population-based longitudinal studies and systematic review Arch Gen Psychiatry 2010 67 9 931 938 20819987 10.1001/archgenpsychiatry.2010.97

[B20] Hodgins S Lapalme M Toupin J Criminal activities and substance use of patients with major affective disorders and schizophrenia: a 2-year follow-up J Affect Disord 1999 55 2–3 187 202 10628888 10.1016/s0165-0327(99)00045-2

[B21] Modestin J Wuermle O Criminality in men with major mental disorder with and without comorbid substance abuse Psychiatry Clin Neurosci 2005 59 1 25 29 15679536 10.1111/j.1440-1819.2005.01327.x

[B22] Swanson JW Swartz MS Essock SM Osher FC Wagner HR Goodman LA Rosenberg SD Meador KG The social-environmental context of violent behavior in persons treated for severe mental illness Am J Public Health 2002 92 9 1523 1531 12197987 10.2105/ajph.92.9.1523 PMC1447272

[B23] Grann M Fazel S Substance misuse and violent crime: Swedish population study BMJ 2004 328 7450 1233 1234 15155501 10.1136/bmj.328.7450.1233 PMC416597

[B24] Boden JM Fergusson DM Horwood LJ Alcohol misuse and violent behavior: findings from a 30-year longitudinal study Drug Alcohol Depend 2012 122 1–2 135 141 22015176 10.1016/j.drugalcdep.2011.09.023

[B25] Fountoulakis KN Leucht S Kaprinis GS Personality disorders and violence Curr Opin Psychiatry 2008 21 1 84 92 18281846 10.1097/YCO.0b013e3282f31137

[B26] Latalova K Prasko J Aggression in borderline personality disorder Psychiatry Q 2010 81 3 239 251 10.1007/s11126-010-9133-3 20390357

[B27] Eckhardt CI Effects of alcohol intoxication on anger experience and expression among partner assaultive men J Consult Clin Psychol 2007 75 1 61 71 17295564 10.1037/0022-006X.75.1.61

[B28] Hines DA Straus MA Binge drinking and violence against dating partners: the mediating effect of antisocial traits and behaviors in a multinational perspective Aggress Behav 2007 33 5 441 457 17683106 10.1002/ab.20196

[B29] Allen A Links PS Aggression in borderline personality disorder: evidence for increased risk and clinical predictors Curr Psychiatry Rep 2012 14 1 62 69 22033830 10.1007/s11920-011-0244-9

[B30] Sansone RA Leung JS Wiederman MW Having been bullied in childhood: Relationship to aggressive behaviour in adulthood Int J Soc Psychiatry 2012 59 8 824 826 22976375 10.1177/0020764012456814

[B31] Silva TC Larm P Vitaro F Tremblay RE Hodgins S The association between maltreatment in childhood and criminal convictions to age 24: a prospective study of a community sample of males from disadvantaged neighbourhoods Eur Child Adolesc Psychiatry 2012 21 7 403 413 22562141 10.1007/s00787-012-0281-x

[B32] Grogan-Kaylor A Otis MD The effect of childhood maltreatment on adult criminality: a tobit regression analysis Child Maltreat 2003 8 2 129 137 12735715 10.1177/1077559502250810

