# Supplementary material for: Risk factors for adult interpersonal violence in suicide attempters
Source: BMC Psychiatry. 2014 Jul 7;14:195. doi: 10.1186/1471-244X-14-195 (PMC4227101; doi:10.1186/1471-244X-14-195)
Supplement: Additional file 1 — The Karolinska Interpersonal Violence Scale. [file 1471-244X-14-195-S1.docx]

**The Karolinska Interpersonal Violence Scale**

**The steps of this scale are defined by short statements about violent behaviour. Based on an interview with the subject; use the highest score where one or more ofthe statements apply.**

**A. Used violence.**

**As a child (6 - 14 years)**

**0** No violence.

**1** Occasional fights, but no cause for alarm among grown-ups in school or in the

family.

**2** Fighter. Been in fights a lot.

**3** Often started fights. Hit a comrade who had been bullied. Continued hitting

when the other had surrendered.

**4** Initiated bullying. Often hit other children, with fist or object.

**5** Caused serious physical injury. Violent toward adult(s). Violent behaviour that

led to intervention by social welfare authorities.

**As an adult (15 years or older)**

**0** No violence.

**1** Slapped or spanked children on occasion. Shoved or shook partner or another

adult.

**2** Occasionally smacked partner or child. Fought when drunk.

**3** Assaulted partner drunk or sober. Repeated corporal punishment of child.

Frequent fighting when drunk. Hit someone when sober.

**4** Instance of violent sexual abuse. Repeated battering/physical abuse of child or

partner. Assaulted/attacked other persons frequently, drunk or sober.

**5** Killed or caused severe bodily harm. Repeated instances of violent sexual abuse.

Convicted of crime of violence.

**B. Victim of violence.**

**Childhood (6 - 14 years)**

**0** No violence.

**1** Occasional slaps. Fights in school, of no great significance.

**2** Bullied occasionally for short period(s). Occasionally exposed to corporal

punishment.

**3** Often bullied. Frequently exposed to corporal punishment. Beaten by drunken

parent.

**4** Bullied throughout childhood. Battered/beaten up by schoolmates. Regularly

beaten by parent or another adult. Beaten with objects. Sexually abused.

**5** Repeated exposure to violence at home or in school that resulted at least once in

serious bodily harm. Repeated sexual abuse, or sexual abuse that resulted in

bodily harm.

**Adulthood (15 years or older)**

**0** No violence.

**1** Threatened or subjected to a low level of violence on at least one occasion.

**2** Beaten by partner on occasion. Victim of purse snatching. Threatened with

object.

**3** Threatened with a weapon. Robbed. Beaten by someone other than partner.

Frequently beaten by partner.

**4** Raped. Battered.

**5** Repeatedly raped. Repeatedly battered. Severely battered, resulting in serious

bodily harm.
